# Assessment of Plasma Tylosin Concentrations: A Comparative Study of Immunoassay, Microbiological Assay, and Liquid Chromatography/Mass Spectrometry

**DOI:** 10.3390/antibiotics12061023

**Published:** 2023-06-07

**Authors:** Eon-Bee Lee, Syed Al Jawad Sayem, Ga-Yeong Lee, Tae-Won Kim, Md Akil Hossain, Seung-Chun Park

**Affiliations:** 1 Laboratory of Veterinary Pharmacokinetics and Pharmacodynamics, College of Veterinary Medicine, Kyungpook National University, Daegu 41566, Republic of Korea; eonbee@knu.ac.kr (E.-B.L.); aljawadsayem@knu.ac.kr (S.A.J.S.); ga7464@naver.com (G.-Y.L.); 2 College of Veterinary Medicine, Institute of Veterinary Science, Chungnam National University, 99 Daehak-Ro, Yuseong-Gu, Daejeon 34134, Republic of Korea; taewonkim@cnu.ac.kr; 3 Department of Oral Biology, College of Dentistry, University of Illinois Chicago, 801 S., Chicago, IL 60612, USA; mdakil@uic.edu; 4 Cardiovascular Research Institute, Kyungpook National University, Daegu 41566, Republic of Korea

**Keywords:** tylosin, plasma concentration, liquid chromatography/mass spectrometry, microbiological assay, enzyme-linked immunosorbent assay

## Abstract

Employing affordable and uncomplicated sample preparation techniques to recommend the most efficient antibacterial therapy could help reduce antibiotic-resistant bacteria. This study evaluated the suitability of immunoassays and microbiological assays as alternatives for liquid chromatography/mass spectrometry (LC/MS) in determining plasma tylosin concentrations after intramuscular administration at a dose of 20 mg/kg to both healthy and diseased pigs in clinical veterinary practice. The diseased pigs were confirmed using the target genes *Actinobacillus pleuropneumoniae* (*apxIVA*) and *Pasteurella multocida* (*kmt1*). The methods showed good linearity, precision, and accuracy. In both healthy and diseased pigs, a significant correlation was observed between LC/MS and the microbiological assay (Pearson correlation coefficient: 0.930, *p* < 0.001 vs. Pearson correlation coefficient: 0.950, *p* < 0.001) and between LC/MS and the enzyme-linked immunosorbent assay (ELISA) (Pearson correlation coefficient: 0.933; *p* < 0.001 vs. Pearson correlation coefficient: 0.976, *p* < 0.001). A strong correlation was observed between the microbiological assay and the ELISA in both healthy and diseased pigs (Pearson correlation coefficient: 0.911; *p* < 0.001 vs. Pearson correlation coefficient: 0.908, *p* < 0.001). A Bland-Altman analysis revealed good agreement between the methods, i.e., 95% of the differences were within the limits of agreement. Therefore, the microbiological assay and the ELISA, which demonstrated sufficient precision and accuracy, can be viable alternatives to LC/MS when it is unavailable.

## 1. Introduction

Tylosin is an antibiotic commonly used in veterinary medicine for the treatment of respiratory infections in pigs [1]. Tylosin belongs to the macrolide class of antibiotics and functions by inhibiting bacterial protein synthesis [2]. Its effectiveness is apparent against Gram-positive bacteria, including *Staphylococcus*, *Streptococcus*, and *Corynebacterium* [3]. Tylosin is commonly employed in veterinary medicine to manage respiratory infections in poultry and livestock and to enhance growth and prevent illness in farm animals when used as a dietary supplement. However, it is currently being used with caution for therapeutic purposes. In particular, it has been used for the prevention and treatment of respiratory diseases caused by the Gram-negative bacterial pathogens *Actinobacillus pleuropneumoniae* and *Pasteurella multocida* in pigs [4,5].

The prevention and treatment of pathogenic respiratory bacterial infections in pigs can be enhanced by directly measuring plasma antibiotic concentration and conducting antibiotic susceptibility testing, and thereby increasing the effectiveness of antimicrobial therapy [6]. In addition, the difference between the minimum inhibitory concentration of tylosin in the media and that in the plasma may affect the determination of appropriate dosage regimens [7]. This suggests that the efficacy of antimicrobial therapy is influenced by factors beyond the intrinsic antimicrobial activity of the antibiotic, and this can be shown by observing the diversity of factors affecting antimicrobial activity in the plasma.

Methods such as liquid chromatography/mass spectrometry (LC/MS), microbiological assay, and enzyme-linked immunosorbent assay (ELISA) can determine plasma concentration [8,9]. These techniques can be utilized alone or in combination with other methods to accurately gauge the plasma concentration of substances, including drugs. A range of factors affect the selection of a method for determining drug concentrations, such as the characteristics of the drug or substance, the accuracy and specificity of the test, and the accessibility of resources and equipment [10]. Alternative analytical methods, such as microbiological assay and ELISA for antibiotics, which are straightforward and cost effective, provide a significant benefit to laboratories lacking specialized and sophisticated equipment [11]. LC/MS analysis is a vital method for the accurate measurement of antibiotic and metabolite concentrations in the blood, and is therefore crucial for drug development and adjusting dosages. However, the use of this method in clinical settings is not feasible for the aforementioned reasons [12]. This highlights the need for on-site analysis. Despite existing methods for on-site analysis, none of them have been confirmed by inter-laboratory studies, and their ability to measure plasma tylosin concentrations is limited [13].

If we can establish a strong correlation between LC/MS and field-applicable microbiological methods or ELISA in healthy and infected swine plasma following the administration of tylosin to pigs, it will be possible to prescribe optimal antibacterial therapies in the field using simple and low-cost sample preparation technology. This could help reduce the emergence of resistant bacteria. Thus, this study aimed to evaluate the correlation of LC/MS with a microbiological assay and an ELISA when determining the plasma concentration of tylosin in healthy and infected pigs.

## 2. Results

### 2.1. Clinical Signs and Target Gene Detection in Pigs

**Figure 1:**
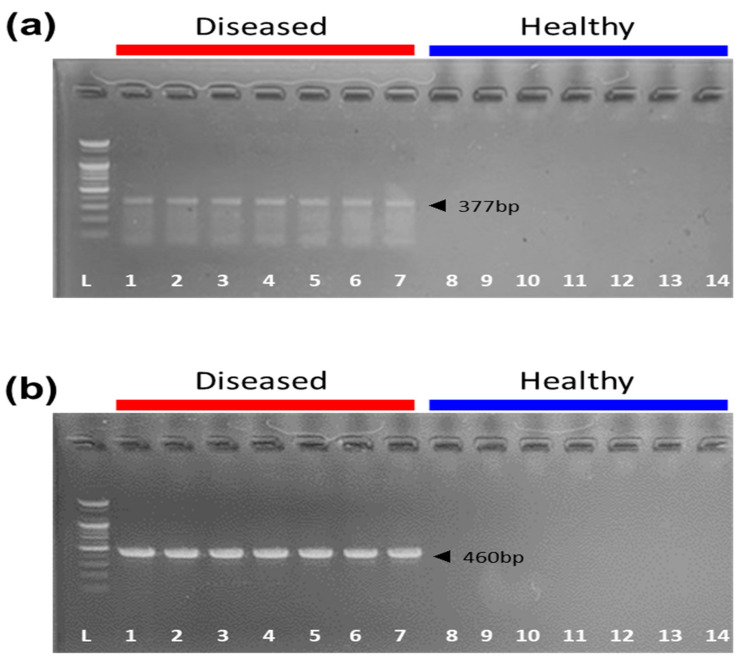
Confirmation of *A. pleuropneumoniae* and *P. multocida* using PCR. (**a**) PCR amplification products with primers for *apxIVA*-targeted *A. pleuropneumoniae*. (**b**) PCR amplification products with primers for *kmt1*-targeted *P. multocida*. Columns 1–7: diseased pigs. Columns 8–14: healthy pigs. L: 100 bp DNA ladder.

Following the successful development of a pig model with coinfections, notable clinical symptoms were observed in comparison with healthy control pigs. We observed a total of 14 pigs (7 healthy, 7 diseased), out of which 5 infected pigs exhibited clinical signs such as reduced activity, labored breathing, and coughing. Similarly, the rest of the infected pigs were lethargic and less engaged with their surroundings compared with their healthy counterparts. The diseased pig samples had the *apxIVA* and *kmt1* polymerase chain reaction (PCR) target genes for *A. pleuropneumoniae* and *P. multocida* with respective fragment sizes of 377 bp (Figure 1a) and 460 bp (Figure 1b), confirming infection in the pigs.

### 2.2. Determination of Plasma Tylosin Concentrations

**Figure 2:**
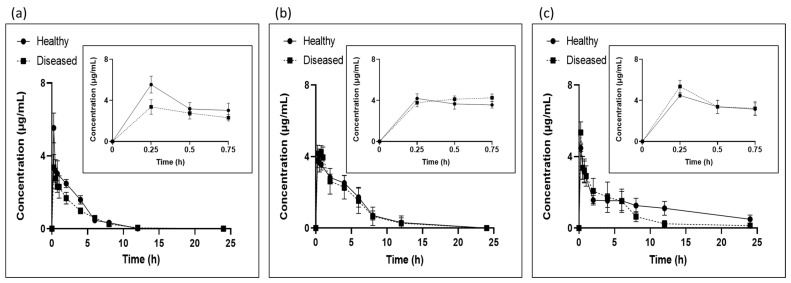
Plasma concentration–time profiles following IM administration of tylosin at a dose of 20 mg/kg, measured using (**a**) LC/MS, (**b**) microbiological assay, and (**c**) ELISA.

The concentration–time profiles of tylosin in the plasma after intramuscular injection in healthy and infected pigs were determined using three methods, namely LC/MS, microbiological assay, and ELISA, and the results are presented in Figure 2.

#### 2.2.1. LC/MS

The tylosin concentration–time profiles obtained using LC/MS are shown in Figure 2a. The linearity of the method was determined using the standard curve of the tylosin concentrations ranging from 0.025 µg/mL to 4 µg/mL. The coefficients of linear regression (r^2^), slope, and y-intercept were 0.98, 1.00, and 0.16, respectively. The limit of detection (LOD) was 0.014 µg/mL and the limit of quantification (LOQ) was 0.042 µg/mL. Both the intra-assay precision and the inter-assay precision were determined to be <20%, and the accuracy of the assays were 101.34–113.62% and 97.38–108.36% for the intra- and inter-assays, respectively (Table 1). The use of blank samples confirmed the absence of interfering peaks in the tylosin-injected samples.

#### 2.2.2. Microbiological Assay

The plasma tylosin concentrations after IM administration analyzed by the microbiological assay are illustrated in Figure 2b. *Micrococcus luteus* was selected as the reference microorganism for the tylosin assay based on the European Pharmacopoeia guidelines [14]. The bacteria are nonpathogenic, grow rapidly at 37 °C, and have good sensitivity to tylosin. To quantify the tylosin concentrations in the plasma samples, standard curves were generated based on the sample matrix. The LOD and LOQ for tylosin in the plasma were both 0.5 µg/mL. The assay was found to be linear between 0.5 µg/mL and 16 µg/mL, with a strong correlation coefficient of R^2^ = 0.99. The intra-assay precision rate ranged from 3.58% to 9.32%, whereas the inter-assay variability was determined by calculating the relative standard deviation (RSD) values from the assays on three different days, and these values ranged from 4.08% to 7.64%. The intra- and inter-assay accuracy rates were 85.95–116.04% and 84.20–102.02%, respectively (Table 2). These results suggest that the microbiological assay met the requirements for quantitative determination in plasma samples. All three analytical methods were confirmed to be usable, as is shown in Figure 2. Therefore, further analysis was conducted to determine the correlation between each of these methods.

#### 2.2.3. ELISA

The plasma tylosin concentrations measured at different time points using the ELISA are described in Figure 2c. The LOD and LOQ for tylosin in the plasma were both 0.005 µg/mL. The intra-assay precision rate was 3.47–12.45%. The inter-assay variability was determined by calculating the relative standard deviation (RSD) values from the assays on three different days, and showed RSD values of 2.67–6.85%. The intra- and inter-assay accuracy rates were 95.41–112.61% and 97.35–108.75%, respectively (Table 3). Thus, the proposed ELISA method for detecting plasma tylosin concentrations was suitable.

### 2.3. Comparison of Methods

#### 2.3.1. Correlation of LC/MS and Microbiological Assay

**Figure 3:**
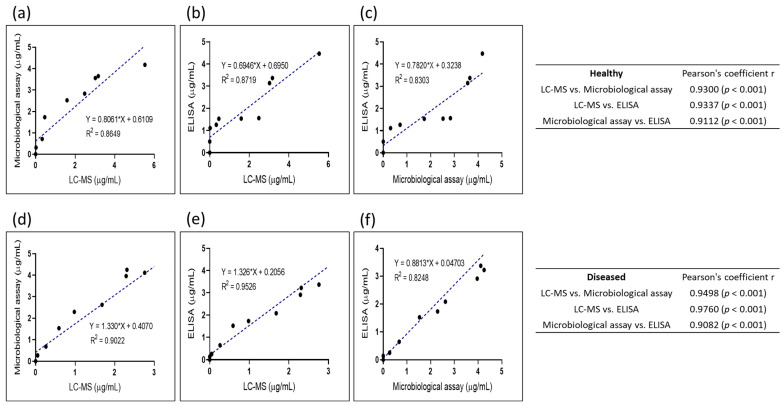
Correlation plot of samples from healthy and diseased pigs treated with tylosin analyzed using LC/MS, microbiological assay, and ELISA. (**a**) Correlation plot of plasma tylosin concentrations obtained using LC/MS and microbiological assay in healthy pigs (r = 0.9300; *p* < 0.001). (**b**) Correlation plot of plasma tylosin concentrations obtained using LC/MS and ELISA in healthy pigs (r = 0.9337; *p* < 0.001). (**c**) Correlation plot of plasma tylosin concentrations obtained using microbiological assay and ELISA in healthy pigs (r = 0.9112; *p* < 0.001). (**d**) Correlation plot of plasma tylosin concentrations obtained using LC/MS and microbiological assay in diseased pigs (r = 0.9498; *p* < 0.001). (**e**) Correlation plot of plasma tylosin concentrations obtained using LC/MS and ELISA in diseased pigs (r = 0.9760, *p* < 0.001). (**f**) Correlation plot of plasma tylosin concentrations obtained using microbiological assay and ELISA in diseased pigs (r = 0.9082; *p* < 0.001).

In the healthy pigs, the correlation between the plasma tylosin concentrations obtained using the LC/MS assay and those obtained using the microbiological assay is presented in Figure 3a. The Pearson correlation coefficient of 0.930 (*p* < 0.001) and the slope of 0.806 indicate a dose-dependent relationship. Likewise, a correlation was observed in the diseased pigs, with a Pearson correlation coefficient of 0.950 (*p* < 0.001) and a slope of 1.330 (Figure 3d).

**Figure 4:**
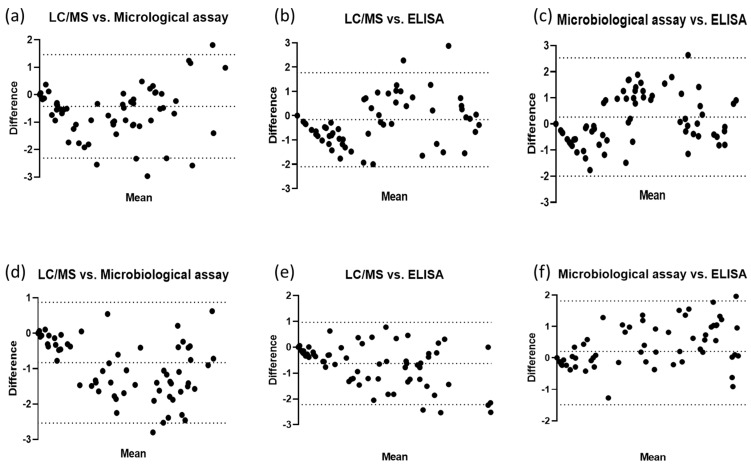
Bland–Altman plots of plasma tylosin concentration determined using LC/MS, microbiological assay, and ELISA with confidence interval limits for mean and agreement limits. (**a**) Bland–Altman plot of LC/MS vs. microbiological assay results for plasma tylosin concentrations in healthy pigs. (**b**) Bland–Altman plot of LC/MS vs. ELISA results for plasma tylosin concentrations in healthy pigs. (**c**) Bland–Altman plot of microbiological assay vs. ELISA results for plasma tylosin concentrations in healthy pigs. (**d**) Bland–Altman plot of LC/MS vs. microbiological assay results for plasma tylosin concentrations in diseased pigs. (**e**) Bland–Altman plot of LC/MS vs. ELISA results for plasma tylosin concentrations in diseased pigs. (**f**) Bland–Altman plot of microbiological assay vs. ELISA results for plasma tylosin concentrations in diseased pigs.

To further evaluate the agreement between the two methods, a Bland–Altman plot was used, and this showed that the mean difference in tylosin concentrations obtained using the two methods was −0.43, with limits of agreement of 2.31 and 1.46 in the healthy pigs (Figure 4a). In the diseased pigs, the mean difference was −0.84, with limits of agreement of −2.54 and 0.87 (Figure 4d). These findings suggest a good agreement between the LC/MS and the microbiological assay for measuring plasma tylosin concentrations.

#### 2.3.2. Correlation of LC/MS and ELISA

Figure 3 shows a dose-dependent relationship between the plasma tylosin concentrations obtained using the LC/MS assay and those obtained using the ELISA in the healthy pigs, with a Pearson correlation coefficient of 0.934 (*p* < 0.001) and a slope of 0.694 (Figure 3b). A significant correlation was also observed in the diseased pigs, with a Pearson correlation coefficient of 0.976 (*p* < 0.001) (Figure 3e).

To assess the agreement between LC/MS and the ELISA, a Bland–Altman plot was produced, and this displayed the differences between the LC/MS and ELISA datasets versus the mean tylosin concentrations obtained using these two methods. The mean difference in the concentrations obtained using the two methods was −0.17 in the healthy pigs, with limits of agreement of −2.10 and 1.77 (Figure 4b), whereas the mean difference in the diseased pigs was −0.63, with limits of agreement of −2.22 and 0.97 (Figure 4e). These results suggest that the LC/MS and ELISA methods are in agreement when used to measure plasma tylosin concentrations.

#### 2.3.3. Correlation of Microbiological Assay and ELISA

A scatter plot in Figure 3 illustrates the correlation between the microbiological coefficient of correlation assay and the ELISA for measuring plasma tylosin concentrations. For the healthy pigs (Figure 3c), a Pearson correlation coefficient of 0.911 (*p* < 0.001) and a slope of 0.782 were observed, whereas for the diseased pigs, a Pearson correlation coefficient of 0.910 (*p* < 0.001) and a slope of 0.881 were observed, indicating a dose-dependent relationship (Figure 3f).

To assess the agreement between the two methods, a Bland–Altman plot was produced, and this displayed the differences between the microbiological assay and ELISA datasets against the mean of the tylosin concentrations obtained using these two methods. The Bland–Altman plot showed good agreement between the microbiological assay and the ELISA for measuring plasma tylosin concentrations; the mean differences in the tylosin concentrations obtained using the two methods were 0.26 in the healthy pigs and 0.20 in the diseased pigs. The limits of agreement were −2.00 and 2.53 in the healthy pigs (Figure 4c) and −1.49 and 1.81 in the diseased pigs (Figure 4f). These results show that both the microbiological assay and the ELISA are rapid, easy, and readily applicable on-site methods that can be used to determine the optimal dose of tylosin.

## 3. Discussion

The more antibiotics are used, the higher the likelihood that antibiotic-resistant bacteria will emerge. This poses a significant problem for public health, as antibiotic-resistant infections are more difficult and costlier to treat. To solve this issue, the One Health Initiative is making efforts to reduce the use of antibiotics in animal farming [15]. This approach recognizes that the health of humans, animals, and the environment are interconnected and aims to address health challenges at the intersection of these areas. Livestock farms differ in the severity of illness they observe, and in their animal breeding practices. Therefore, antibiotics must be used prudently and only when necessary. When administering antibiotics to pigs, the amount of antibiotics present in their blood should be monitored because the optimal dosage required to treat an infection can depend on factors such as the pig’s weight and disease severity.

High-performance liquid chromatography (HPLC) and LC/MS are the most widely accepted methods of assessing drug plasma concentrations, and they are both highly accurate and capable of analyzing multiple compounds in a single sample [16]. However, owing to their high cost and maintenance requirements, most laboratories do not have access to these methods [17]. Consequently, a cost-effective technique for measuring antibiotics in the plasma or blood that can be used on livestock farms is needed.

In this study, we aimed to develop a low-cost method for determining plasma tylosin concentrations that can be used in laboratories where LC/MS is inaccessible. Alternative methods for measuring drug concentrations, such as immunoassays and microbiological assays, are available [18,19]. Although HPLC and LC/MS are both valid options, previous studies have not explored the potential use of microbiological assays and ELISAs to measure plasma tylosin concentrations.

Comparing microbiological and instrumental methods with other biological methods is a common practice used to investigate potential variations in the pharmacological activity of drugs and determine drug concentrations in the plasma. It has been found that microbiological assays may have limited sensitivity compared with other analytical methods. The detection limit of the assay may not be sufficient to accurately measure low drug concentrations in plasma samples. This can be a limitation when analyzing drugs that exhibit low plasma concentrations, or when studying drug pharmacokinetics in the body [20]. A previous study revealed significant correlations between microbiological and LC/MS methods for measuring the plasma concentrations of cefquinome, cefotaxime, meropenem, and piperacillin [20]. However, discrepancies were found in the plasma concentrations of clarithromycin [21]. This could be because of the active clarithromycin metabolites present in the bloodstream. This metabolite, known as 14-OH-clarithromycin, has been found to possess significant antibacterial properties against certain Gram-negative pathogens. Although microbiological assays and LC/MS generally exhibit a high correlation, some differences exist between these two methods, possibly because lower concentrations are found in the microbiological assay due to antibiotic plasma degradation [22,23].

In this study, we analyzed plasma tylosin concentrations over time in healthy and diseased pigs and found similar concentration changes over time using all three methods. Each method demonstrated satisfactory performance with inter- and intra-assay coefficients of variation within ±20.0% for the RSD (%). These results suggest that the microbiological and ELISA methods may be used in place of LC/MS for measuring tylosin, and that they have potential for rapid and on-site application. To confirm this possibility, we statistically analyzed the data to compare the three methods.

Bland and Altman developed the Bland–Altman plot to evaluate similarities between two sets of numerical measurements. In this study, the Bland–Altman plot indicated a high level of agreement between the measurement methods, with 95% of the differences between the two methods falling within the appropriate limits. However, the suitability of these limits should be determined based on clinical and biological considerations [10]. To use a Bland–Altman plot effectively, appropriate limits must be set based on relevant criteria, and whether these limits are exceeded must be assessed using statistical analysis [24]. A linear regression analysis can also be used to predict one variable based on another, and to quantify the degree of fit using the coefficient of correlation (r) [25]. In this study, the methods used for tylosin measurement showed very good agreement, as is indicated by the regression lines between LC/MS and the microbiological assay (r = 0.9300; *p* < 0.001), between LC/MS and the ELISA (r = 0.9337; *p* < 0.001), and between the microbiological assay and the ELISA (r = 0.9112; *p* < 0.001) in the healthy pigs, and by those between LC/MS and the microbiological assay (r = 0.9498, *p* < 0.001), between LC/MS and the ELISA (r = 0.9760; *p* < 0.001) and between the microbiological assay and the ELISA (r = 0.9082; *p* < 0.001) in the diseased group.

Although the ELISA is a valuable tool, there are certain limitations and obstacles that may affect the precision and consistency of its results [26]. Its specificity relies on the ability of antibodies to identify particular target molecules; however, these antibodies may also bind to molecules that have similar structures, leading to inaccurate positive results [27]. Furthermore, ELISAs may have a limited capability to detect molecules that are present in low amounts in complex samples such as plasma, and thus generate false-positive results [28]. The accuracy of an ELISA test is heavily reliant on various assay conditions that can influence its sensitivity and specificity, such as temperature, pH, and incubation time [29].

This study found good correlation between different analytical methods used to measure tylosin levels in plasma. ELISAs and microbiological assays have several advantages, including simplicity, cost-effectiveness, and the ability to provide important insights into a drug’s effectiveness and potency. However, it is important to acknowledge that these assays may not be suitable for all types of drugs, and their limitations should be taken into account. Certain drugs may not be accurately detected or quantified by these assays due to factors such as interference from other substances, complex drug formulations, or the absence of specific microbial targets. Therefore, while microbiological assays can be valuable in drug testing, it is crucial to supplement them with other analytical methods to ensure a thorough and precise evaluation of a drug’s properties.

This study involved creating a diseased porcine model through intranasal inoculation. The pigs infected with *A. pleuropneumoniae* and *P. multocida* displayed various symptoms, including anorexia, high body temperature, labored breathing, and coughing, all of which are symptoms that are typically observed in diseased pigs [30]. After the clinical signs were observed, further experiments were conducted on these pigs. The physiological changes brought about by an infection can affect pharmacokinetics [31]. Therefore, the pharmacokinetic properties of the diseased pigs may have been different from those of healthy animals [32]. Hence, it was determined that studying the concentration–time profiles of tylosin in the diseased pigs and comparing them with those of healthy pigs would be beneficial for the clinical application of antibacterial therapy.

## 4. Materials and Methods

### 4.1. Chemicals and Reagents

Standard tylosin (>96%) was purchased from Sigma (St. Louis, MO, USA). The injectable tylosin was obtained from Samyang Anipharm (Seoul, Korea). Nicotinamide adenine dinucleotide (NAD) was obtained from Sigma. All chemicals and reagents used in this study were of HPLC grade.

### 4.2. Bacterial Strains

*A. pleuropneumoniae* (BA2000013) and *P. multocida* (BA1700127) were provided by the Animal and Plant Quarantine Agency (Gimchen, Korea). Before use, the bacterial isolates were streaked on brain heart infusion agar (Becton, Dickinson and Company, Franklin Lakes, NJ, USA) supplemented with 0.02% NAD and incubated at 37 °C in 5% CO_2_. For the microbiological assay, *M. luteus* KCCM 11236 was obtained from the Korean Culture Center of Microorganisms (KCCM) and cultured on tryptic soy broth (Becton, Dickinson and Company, Franklin Lakes, NJ, USA) at 37 °C for 18 h.

### 4.3. Animal Experiment

#### 4.3.1. Experimental Design

The study was carried out on 14 pigs (Duroc × Landrace × Yorkshire) aged 5–6 weeks with an average weight of 9.5 ± 1.1 kg. The animals were acclimatized for 1 week with free access to the water and feed. The animal study was approved by the Animal Ethics Committee of the Petobio Clinical Institute (PTB-2022-IACUC-013-A). After proper adaptation, the pigs were divided into a healthy group and an infected group, with each group consisting of seven animals. The bacterial challenge was performed according to a previously described method, but with some modifications [33]. The 40 mL bacterial suspension was subjected to centrifugation at 3500 rpm for 10 min, after which the supernatant was removed. The bacterial cells were then suspended again in 40 mL of 0.9% NaCl solution. The pigs in the diseased group were intranasally inoculated with a 1 mL mixture of 2.0 × 10^9^ cfu/mL of *A. pleuropneumoniae* and *P. multocida* to establish a disease model. During the experiment, clinical respiratory disease score, intestinal function score, appearance/abnormal signs, and clinical signs were recorded [34,35]. *A. pleuropneumoniae* and *P. multocida* infections were monitored by culturing nasal swabs, and infections were confirmed by the presence of the *apxIVA* gene [36] and the *kmt1* gene [37], used for detecting *A. pleuropneumoniae* and *P. multocida*, respectively.

#### 4.3.2. Blood Collection

The healthy and diseased pigs received intramuscular tylosin injections at a dose of 20 mg/kg. The selection of a 20 mg/kg drug dose was based on previous studies [38] that had shown this dose to be within the therapeutic range for the target drug and species under investigation. This dosage was chosen to ensure that the drug concentration in the plasma samples would fall within a quantifiable range, allowing for accurate and reliable analysis. Blood samples were collected from the jugular vein at 0 h, 0.25 h, 0.5 h, 0.75 h, 1 h, 2 h, 4 h, 6 h, 8 h, 12 h, and 24 h. The blood samples were centrifuged at approximately 3000× *g* for 10 min at approximately 5 °C. The plasma from each Vacutainer was divided into aliquots of about 0.6 mL which were deep frozen at approximately −75 °C until analysis.

#### 4.3.3. Plasma Sample Processing for LC/MS Analysis

Preparation for the LC/MS analysis was as follows. The 250 μL plasma samples were thawed at room temperature and then deproteinized with methanol (2 mL) via vortexing for 15 min and centrifuged at 3300× *g* for 10 min at 4 °C. The 2 mL of supernatant was collected to dryness in a water bath using nitrogen at 50 °C. The residue was then dissolved in 200 μL of methanol, agitated for 1 min, and centrifuged at 12,000 rpm for 10 min at 4 °C. The final 70 μL of supernatant was analyzed using LC/MS.

### 4.4. Analysis of Plasma Tylosin Concentrations

#### 4.4.1. Microbiological Assay

Plasma tylosin concentrations were determined via a microbiological assay using *M. luteus* KCCM 11236 as the test organism [39]. The bacterial suspension obtained from overnight growth in tryptic soy broth was adjusted to an optical density of 0.5 at 600 nm. Subsequently, the *M. luteus* KCCM 11236 bacterial suspension was added to tempered nutrient agar from Becton, Dickinson and Company, Franklin Lakes, NJ, USA, at a concentration of 10^6^ cfu/mL. The agar mixture was immediately poured onto assay plates in 2.2 mm layers. After allowing the samples to solidify for 45 min, wells with 0.5 cm diameters were created and filled with 60 μL plasma samples or tylosin standards covering a range of concentrations from 1 μg/mL to 16 μg/mL. The agar plates were then incubated for 18 h at 37 °C. The zones of bacterial inhibition were measured using a digital caliper from Mitutoyo, Japan. This method was chosen for its high sensitivity, simplicity, and strong correlation with HPLC measurements [40]. The method was validated according to a previously described process [41].

#### 4.4.2. ELISA

Plasma tylosin concentrations were assessed using an ELISA kit (E-FS-E058) obtained from Amsbio (Abingdon, Oxfordshire, UK). A 1 mL serum sample was transferred to a 2 mL e-tube and centrifuged for 5 min at 4000 rpm at room temperature. Following centrifugation, 0.1 mL of the supernatant was collected and mixed with 0.9 mL of the reconstitution buffer provided by the ELISA kit. The mixture was then oscillated for 30 s. Finally, a 50 μL sample was extracted for detection, following the manufacturer’s instructions. The optical density was determined at 450 nm using an Epoch microplate reader (Winooski, VT, USA). The percentage of absorbance was determined using the following formula: (A/A_0_) × 100%. Here, A = average absorbance of standard or sample and A_0_ = Average absorbance of 0 ppb standard. For the standard curve, the absorbance percentage of each standard was plotted on the *y*-axis, and the logarithmic concentration was plotted on the *x*-axis, resulting in a semi-log plot. The precision of the assays was evaluated by assessing repeatability, which was expressed as the relative standard deviation (RSD). To determine accuracy, a control sample was introduced at the beginning of the procedure, and the measured value was calculated by dividing it by the nominal value and multiplying the result by 100.

Tylosin has a low metabolic rate after administration, and its residues are widely distributed in the body fluids and tissues of livestock [42]. However, antibiotically potent metabolites commonly found after the administration of tylosin to swine include desmycosin (tylosin B), macrosin (tylosin C), relomycin (tylosin D), dihydrodesmycosin, and at least 10 other degradates in smaller quantities [43]. According to the manufacturer’s data sheet, the cross-reactivity of the specific antibody with tylosin and its active metabolites is 100%, whereas the cross-reactivity of erythromycin and other macrolide antibiotics are 1% and <1%, respectively. These data indicate the high specificity of this ELISA kit and the low possibility that interactions of specific antibodies with tylosin metabolites will influence the assay result.

#### 4.4.3. LC/MS Analysis

The LC/MS analysis was performed using a 1200 HPLC system from Agilent Technologies (Santa Clara, CA, USA) that was connected to a 6140 single mass spectrometer (Agilent Technologies). The mass spectrometer was set up with an electrospray positive ionization mode using a capillary voltage of 3500 V, and it had optimal ESI-MS parameters, such as a drying gas temperature of 350 °C, a drying gas flow of 5 L/min, and a nebulizing gas pressure of 45 psi. The separations were accomplished using an Eclipse Plus C18 column (2.1 × 100 mm, 3.5 μm) from Agilent Technologies. The mobile phase consisted of a mixture of 0.1% formic acid in water (Eluent A) and 0.1% formic acid in acetonitrile solution (Eluent B), with a ratio of 30:70 (*v*/*v*). The flow rate was 0.4 mL/min and the sample injection volume was 3 μL. The column temperature was maintained at 40 °C. The monitored precursor ion for tylosin was 916.3 *m*/*z*. The validation of the assay was performed according to a procedure described elsewhere [42]. To conduct the chromatographic analysis, initial standard solutions for each analyte were prepared by dissolving them in a solvent with the same composition as that of the mobile phase. These stock standard solutions had a concentration of 0.1 μg/mL. For daily use, working standards were freshly prepared. To establish the calibration curves, a series of solutions were prepared at various concentrations ranging from 0.025 μg/mL to 4 μg/mL. These solutions were used to plot the calibration curves, which served as references for determining the concentrations of the analytes in the samples. The suggested method was assessed using spiked plasma samples at five different levels. The LOD was the concentration at which the signal-to-noise ratio was greater than 3, while the LOQ was the concentration at which the signal-to-noise ratio was greater than 10 in the blank samples spiked with the analytes. Tylosin concentrations in the plasma samples from the infected and non-infected animals were determined using this validated LC/MS method.

### 4.5. Statistical Analysis

Statistical analyses, including data processing and graph creation, were conducted using GraphPad Prism 8.0 (GraphPad Software Inc., San Diego, CA, USA). The agreement of the three analytical methods was evaluated using the Pearson correlation coefficient method and the Bland-Altman method [25,43].

## 5. Conclusions

In this study, the results of comparing three different methods for measuring tylosin plasma concentrations were significant, considering the importance of monitoring antibiotic concentrations in animals to prevent the emergence of antibiotic-resistant bacteria, which can be caused by the overuse of antibiotics in farming practices. Thus, the microbiological assay and the ELISA, both of which are cost effective and accessible, could replace LC/MS. Nevertheless, these simpler methods still require improvements in their precision and consistency before they can be extensively used in clinical applications for antibacterial therapy.

## Figures and Tables

**Table 1:** Intra-assay and inter-assay variations in LC/MS.

Assays		Nominal Concentration (µg/mL)				
		4	1	0.1	0.05	0.025
Intra-assay	Mean concentration (n = 5)	4.06	1.04	0.11	0.05	0.03
	Precision (RSD, %)	0.95	2.13	13.63	8.34	16.90
	Accuracy (%)	101.39	104.21	113.62	101.34	101.56
Inter-assay	Mean concentration (n = 5)	4.08	0.97	0.11	0.05	0.03
	Precision (RSD, %)	1.94	11.84	14.91	16.15	20.05
	Accuracy (%)	102.03	97.38	108.36	101.85	105.77

LC/MS: liquid chromatography/mass spectrometry; RSD: relative standard deviation; n: number of observations.

**Table 2:** Intra- and inter-assay variations in the microbiological assay.

Assays		Nominal Concentration (μg/mL)				
		16	8	4	2	1
Intra-assay	Mean concentration (n = 5)	14.41	8.38	3.44	2.32	0.90
	Precision (RSD, %)	9.32	8.16	4.45	6.36	3.58
	Accuracy (%)	90.08	104.76	85.95	116.04	89.99
Inter-assay	Mean concentration (n = 5)	14.25	8.16	3.37	2.32	0.90
	Precision (RSD, %)	7.64	6.95	5.54	6.59	4.08
	Accuracy (%)	89.09	102.02	84.20	115.91	89.59

RSD: relative standard deviation; n: number of observations.

**Table 3:** Intra- and inter-assay variations in ELISA.

Assays		Nominal Concentration (μg/mL)				
		40.5	13.5	4.5	1.5	0.5
Intra-assay	Mean concentration (n = 5)	42.35	12.88	4.48	1.47	0.56
	Precision (RSD, %)	4.92	3.47	10.71	12.45	7.20
	Accuracy (%)	104.57	95.41	99.50	97.77	112.61
Inter-assay	Mean concentration (n = 5)	42.17	13.14	4.62	1.52	0.54
	Precision (RSD, %)	5.74	2.67	4.88	2.93	6.85
	Accuracy (%)	104.12	97.35	102.77	101.13	108.75

RSD: relative standard deviation; n: number of observations.

## Data Availability

Data are available on reasonable request from the corresponding author.
